# Composite aortic root replacement in African patients with type A aortic dissection: report of 12 cases

**DOI:** 10.11604/pamj.2023.45.18.37147

**Published:** 2023-05-05

**Authors:** Charles Mve Mvondo, Laurence Carole Ngo Yon, Hermann Nestor Tsague Kengni, Marcelin Ngowe Ngowe

**Affiliations:** 1 Division of Cardiac Surgery, Cardiac Center of Shisong, St Elizabeth Catholic General Hospital, Kumbo, Cameroon,; 2 Department of Surgery, Faculty of Medicine and Pharmaceutical Sciences, Douala, Cameroon,; 3 Department of Cardiology, Jordan Medical Services, Yaoundé, Cameroon,; 4 Department of Cardiology, National Social Insurance Fund Hospital, Yaoundé, Cameroun,; 5 Department of Internal Medicine, Faculty of Medicine and Biomedical Sciences, Yaoundé, Cameroon

**Keywords:** Acute type A aortic dissection, composite root replacement, sub-Saharan Africa

## Abstract

Type A aortic dissection (TAAD) is associated with high mortality in the absence of appropriate surgical therapy. The involvement of the aortic root by the intimal tear and the presence of severe aortic insufficiency will require a more radical approach with composite root replacement (CRR) in most of the patients. We briefly report our surgical experience following CRR in 12 patients presenting with TAAD in our department. Between November 2009 and January 2022, a total of twelve (n=12) patients diagnosed with TAAD were operated in our institution. Clinical data and surgical outcomes were retrospectively reviewed. The mean age at admission was 51.1 ± 12.43 years (range: 34-72). One patient met the criteria for Marfan´s disease (1/12, 8.3%). The operative mortality was 16.66% (2/12). Composite root replacement with a mechanical valved conduit was performed in the majority (11/12, 91.66%;) whereas a separated supracoronary graft replacement and aortic valve replacement were performed in one patient. Concomitant aortic arch surgery (hemi or total) was done in 9/12 patients (75%). The commonest postoperative complications were: chest re-exploration for bleeding in 2/12 (16.66%), transitory cerebral ischemia in 1/12 (8.33%) and low cardiac output syndrome in 2/12 (16.66%). The mean length of stay in the Intensive Care Unit (ICU) was 4.8±3.8 days (range: 2-17). Delayed referral of patients with TAAD was observed in the majority of patients as they were operated in the subacute or chronic phase. Composite root replacement in these patients is associated with acceptable outcomes despite complex anatomic-pathological lesions.

## Introduction

Stanford's type A aortic dissection (TAAD), characterized by the involvement of the proximal aortic segment by an intimal tear, is associated with high mortality when left untreated [1,2]. In the sub-Saharan region (SSA), the prevalence of TAAD remains unclear, while the availability of prompt curative repair is limited by poor access to diagnostic facilities and specialized cardiovascular care [3,4]. In our recent series, thoracic aneurysms were mainly diagnosed in their late course and presented with challenging anatomy-pathological lesions requiring extensive and complex repair such as composite root replacement (CRR) [5]. Although CRR is a well-established technique in aortic surgery in experienced centers, it might appear technically demanding in low-volume centers, carrying an increased operative risk, especially in the setting of emergency TAAD repair. The current paper reviews our experience with TAAD surgery, where most patients had undergone CRR surgery.

## Methods

Twelve (n=12) patients diagnosed with Stanford type A aortic dissection underwent surgery at the Division of Cardiac Surgery at the Shisong Cardiac Center between July 2014 and January 2022. Their clinical records were retrospectively reviewed to analyze their demographic and clinical profiles, as well as the surgical outcomes related to composite root replacement interventions. Patient characteristics are summarized in Table 1.

**Table 1:** patient’s demographic and clinical profiles

Variables	Value (Total: 12 patients)
Age in years, mean ± SD (range)	51.1 ±12.43 (34-72)
Sex ratio (M/F)	1.16
BSA, mean ± SD (range)	1.92 ± 0.2 (1.5-2.3)
BMI, mean ± SD (range)	26.6±5.2 (19.05-35.5)
Hypertension, n (%)	11 (91.6)
Smoking, n (%)	3 (25)
Genetic syndromes, n (%)	1 (8,3)
Acute aortic dissection (< 2 weeks), n (%)	4 (33.3)
**Symptoms, n (%)**	
History of chest pain	11 (91.6)
Dyspnea > NYHA class III	8 (66.6)
**Associated lesions, n (%)**	
Aortic valve insufficiency Mitral insufficiency	12 (100) 1 (8.3)
Left ventricle ejection fraction < 50%, n (%)	1 (8.3)
HIV serology, n (%)	1 (8.3)
Impaired renal function at admission, n (%)	2 (16.6)
Malperfusion syndrome at admission, n (%)	3 (25)

BSA: body surface area, BMI: body max index, NYHA: New York Heart Association, HIV: Human Immunodeficiency Virus

**Figure 1:**
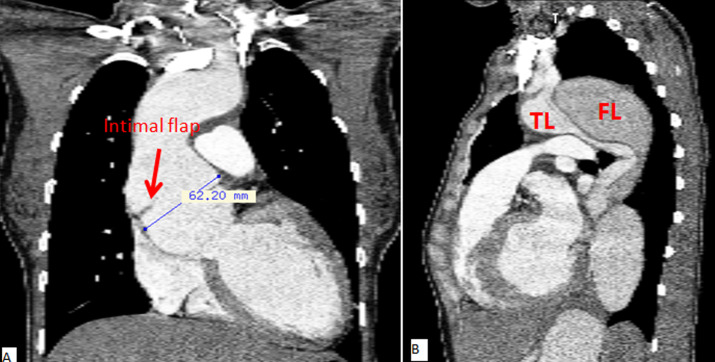
A,B) preoperative computed tomography angiography

**Preoperative assessment and indication for surgery:** the majority of patients were referred from other hospitals and/or physicians following a TAAD diagnosis by computed tomography angiography (Figure 1). Routine transthoracic echocardiography was performed during admission in all patients to rule out threatening conditions, such as tamponade, and to assess key cardiac parameters (ventricular dimensions, contractility, pulmonary hypertension, valvular function, etc.). A team of cardiac surgeons, cardiologists, and anesthesiologists decided to perform the surgical repair in each case. Considering the high-risk procedure and socio-cultural nature of our environment, patients and families were equally involved to ensure a clear understanding of the risks and benefits of the planned surgery.

**Surgical technique:** chest opening was performed through a full median sternotomy in all cases. The right axillary artery was the preferred arterial cannulation site, followed by the innominate artery and the distal aortic arch (when the intimal flap was limited to the ascending segment). Venous drainage was obtained through bicaval cannulation to facilitate both the external removal of the infused crystalloid cardioplegia from the right atrium and retrograde cerebral perfusion through the superior venous cannula if needed. Transoesophageal echocardiography was routinely used in all cases for intraoperative assessment of aortic disease and associated lesions and to support the planning of the surgical strategy. Crystalloid cold cardioplegia (Custodiol HTK; Köhler Chemie GmbH, Bensheim, Germany) was used for myocardial protection in all patients and was administered selectively in the coronary ostia. In the absence of associated disease, a proximal procedure (CRR or aortic valve replacement) was performed first. In cases that required aortic arch repair, the open technique was preferred for distal anastomosis. This was performed under deep hypothermic arrest (mean rectal temperature, 26-28°C) with antegrade selective cerebral perfusion through one or both carotid arteries (Kazui´s technique).

**Statistical analysis:** statistical analysis was performed with StatView 4.5 (SAS Institute Inc., Abacus Concepts, Berkeley, CA). Continuous variables were expressed as mean ±1 standard deviation, whereas categorical variables were presented as absolute numbers and percentages.

## Results

**Figure 2:**
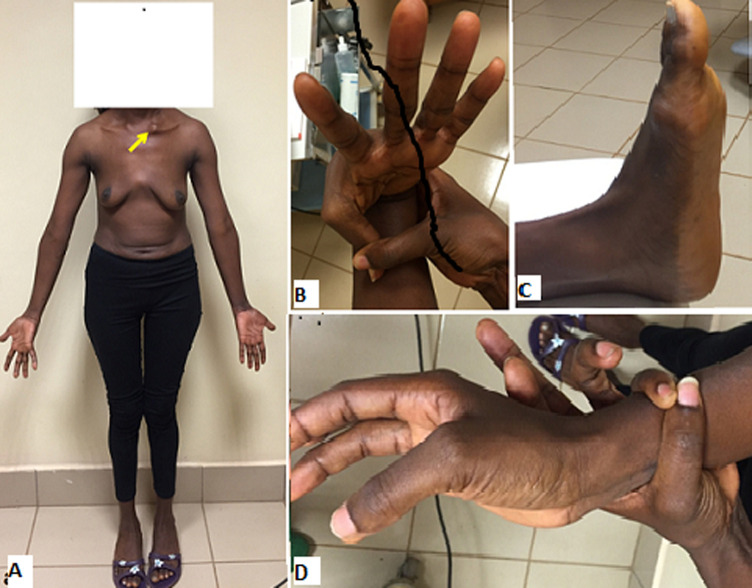
A,B,C,D) patient with Type A aortic dissection and suspicion of Marfan’s disease: chronic non-traumatic left clavicle dislocation

**Figure 3:**
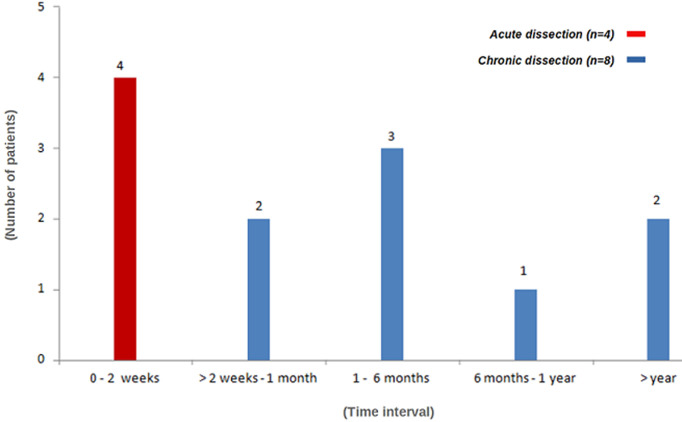
timing of surgical repair from the onset of symptoms (based on patient history)

**Profiles of the patients:** there was no significant difference in occurrence of TAAD between males and females. The mean age of the whole cohort was 51.1±12.43 years (range: 34-72 years). The most common risk factor was uncontrolled hypertension in 11/12 (91.6%) patients. Connective tissue disorder was diagnosed in one patient (8.3%) who met the criteria for Marfan´s disease (Figure 2). Six patients (6/12, 50%) were overweight (BMI ≥ 25 to < 30) whereas two were obese (2/12, 16.6%) (BMI > 35). Most patients had a history (past or recent) of acute chest pain (91.6%). New York Heart Association (NYHA) class dyspnea was present in 8/12 (66.6%) patients. Finally, eight (8/12; 66.6%) patients were referred in the chronic phase (<2 weeks) following the onset of TAAD symptoms and diagnosis (Figure 3).

**Preoperative echocardiography data:** an intimal flap was clearly visualized in the ascending segment in the majority (11/12) cases (Figure 1). This was not the case in one patient in whom proximal thrombosis of the false lumen was confirmed intraoperatively. Moderate-to-severe aortic insufficiency was present in all patients, and 58.3% (7/12) had primary aortic valve disease. One patient had associated severe functional mitral insufficiency resulting from a dilated left ventricle, and one patient was diagnosed with a left ventricular ejection fraction of <50%. Three patients (3/12, 25%) had moderate pericardial effusion at admission without hemodynamic instability.

**Surgical outcomes:** the operative mortality rate was 16.6% (2/12). Composite root replacement with a mechanical valved conduit was performed in most patients (91.66%; 11/12), whereas one patient underwent separate supracoronary graft repair and aortic valve replacement. Concomitant aortic arch surgery (hemi or total) was done in 9/12 patients (75%). The mean cardiopulmonary and aortic cross-clamping times were 270 ± 78.1 min and 189.8 ± 39.6 min, respectively. The most common postoperative complications were chest re-exploration for bleeding 2/12 (16.66%), transitory cerebral ischemia 1/12 (8.33%), and low cardiac output syndrome 2/12 (16.66%). The mean intensive care unit length of stay was 4.8 ± 3.8 days. Operative data are summarized in Table 2.

**Table 2:** operative data

Variable	Value (Total: 12 patients)
Composite root replacement (Bentall), n (%)	11 (91.6)
Supracoronary graft replacement, n (%)	1 (8.3)
Arterial cannulation, n (%)	
Right axillary artery	10 (83.3)
Distal arch	1 (8.3)
Innominate artery	1 (8.3)
Mechanical aortic valve implantation, n (%)	11 (91.6)
Associated procedures (non aortic), n (%)	
Mitral repair	1 (8.3)
Aortic arch surgery (hemi or total arch), n (%)	9 (75)
CPB time in min, mean ± SD (range)	270 ± 78.1 (157-405)
Cross Clamping time in min, mean ± SD (range)	189.8 ±39.6 (135-260)
Complications, n (%)	
Bleeding	2 (16.6)
Stroke	1 (8.3)
LCO syndrome	2 (16.6)
ICU length of stay in days, mean ± SD (range)	4.8±3.8 (2-17)
Overall hospital mortality, n (%)	2 (16.6)
Causes of deaths, n (%)	
Postoperative malperfusion syndrome	2 (16.6)

CPB: cardiopulmonary bypass; LCO: low cardiac output syndrome; ICU: intensive care unit

## Discussion

Compared to distal aortic dissection, the involvement of the aortic root is associated with an increased risk of life-threatening cardiovascular events, such as myocardial ischemia, acute aortic insufficiency, stroke, and cardiac tamponade [6,7]. Indeed, root replacement procedures with CRR or valve-sparing procedures are recommended to reduce the rate of major cardiac complications, including the risk of reoperation in patients with dissected aortic root [8-10]. More generally, the surgical technique in TAAD is dictated by the anatomy of the lesions, with supra coronary graft repair (SGR) with or without aortic valve replacement being the most common procedure [9]. Furthermore, SGR is a simpler technique requiring a shorter cardiopulmonary bypass (CPB) time, which might favor its use in emergency situations where the primary goal is to save the patient´s life. However, no advantage in terms of complication rate was reported by the International Registry of Aortic Dissection (IRAD) when comparing SGR with root replacement techniques [11]. The data of 1,995 patients enrolled in the registry showed no significant differences in operative mortality rates between the two techniques. However, increased cardiac events, such as myocardial ischemia, were reported in the SGR group despite shorter CPB and cross-clamping times. Thus, the surgical technique in TAAD should consider not only the anatomical lesions or the patient´s clinical status but also factors such as the surgeon´s and institution´s expertise, including the possibility of providing hybrid procedures. Valve-sparing surgery in TAAD patients might be time-consuming in less experienced hands, increasing the operative risk, whereas satisfactory results could be obtained in complex aortic lesions in centers providing multidisciplinary or hybrid interventions [12-14]. In regions with limited cardiovascular institutions, such as developing countries, the management of patients with TAAD is challenging as there is still a poor clinical interest in this “neglected” disease, as reflected by the paucity of scientific reports [15]. Despite the fact that the risk factors for aortic diseases such as endemic infections [16,17], and the world´s highest prevalence of hypertension (33%-60%) [11-13,18-20] have been reported in SSA populations, few studies on aortic aneurysm incidence and therapeutic management have been conducted [21-23]. In our recent experience, we found that thoracic aortic aneurysms and TAAD occurred at a younger age in SSA patients than in Western series, with more than 60.7% reporting a history of uncontrolled hypertension [5]. Moreover, the diagnosis was mostly incidental, and many patients diagnosed with chronic TAAD had stable clinical conditions that allowed elective repair with more extensive surgical resection. Thus, composite root replacement was the main procedure in our TAAD patients (91.6%), whereas only one patient underwent SGR. The choice of CRR was dictated by the relatively young age of the patients, the presence of a dissected or dilated root, and a primary disease of the aortic valve, which contraindicated valve-sparing approaches.

The most common surgical challenges were related to the lack of equipment, mainly because of the institution´s financial constraints. To some extent, the lack of a near-infrared spectroscopy monitoring system has made selective cerebral perfusion maneuvers hazardous. Second, the unavailability of hybrid devices did not permit a more distal intervention, which could have been performed in some cases (frozen elephant trunk). Other factors were related to the patient, such as the potential impact of suboptimal anticoagulation intake on both prosthetic valve-related events and the long-term patency of the false lumen. Lastly, the volume of aortic aneurysm surgery is still relatively poor in our institution, this has potentially affected the experience of the local team. The surgical outcomes in our study were influenced by a large number of chronic lesions. When compared with acute TAAD, chronic TAAD repair seems to be associated with better results in terms of operative mortality rates ranging between 4.5% and 11.3% in some series [24,25]. Indeed, the current study, including 66% of patients with chronic TAAD, reported an operative mortality rate of 16.6%, which compares favorably with data from international registries in acute TAAD, ranging between 19.8% and 21.3% [11,26]. Our results were better than previous SSA experiences in which TAAD patients were conservatively managed [27,28].

**Limitations:** of the current study include the small sample size of the cohort and the retrospective nature of our analysis.

## Conclusion

In conclusion, patients with TAAD presented with a history of uncontrolled hypertension. Delayed diagnosis and late referral were the rules, as most were operated on in the sub-acute or chronic phase of TAAD with clinically stable conditions. Thus, a more radical resection with composite root replacement was feasible in nearly all patients, with acceptable early surgical results.

### What is known about this topic

- *Stanford type A aortic dissection (TAAD) is associated with high mortality in the absence of appropriate surgical therapy; composite aortic root replacement is recommended to reduce the rate of major cardiac complications*.

### What this study adds

- *In the sub-Saharan region (SSA), the prevalence of TAAD remains unclear, even if the availability of prompt curative repair is limited by poor access to diagnostic facilities and specialized cardiovascular care, a more radical resection with composite root replacement is feasible in our context, with acceptable early surgical results*.
